# Glucose Concentrations from Continuous Glucose Monitoring Devices Compared to Those from Blood Plasma during an Oral Glucose Tolerance Test in Healthy Young Adults

**DOI:** 10.3390/ijerph182412994

**Published:** 2021-12-09

**Authors:** Thomas G. Kontou, Charli Sargent, Gregory D. Roach

**Affiliations:** The Appleton Institute for Behavioural Science, Central Queensland University, P.O. Box 42, Adelaide 5034, Australia; charli.sargent@cqu.edu.au (C.S.); greg.roach@cqu.edu.au (G.D.R.)

**Keywords:** continuous blood glucose monitoring, blood glucose, healthy participants

## Abstract

Continuous glucose monitoring devices measure glucose in interstitial fluid. The devices are effective when used by patients with type 1 and 2 diabetes but are increasingly being used by researchers who are interested in the effects of various behaviours of glucose concentrations in healthy participants. Despite their more frequent application in this setting, the devices have not yet been validated for use under such conditions. A total of 124 healthy participants were recruited to a ten-day laboratory study. Each participant underwent four oral glucose tolerance tests, and a total of 3315 out of a possible 4960 paired samples were included in the final analysis. Bland–Altman plots and mean absolute relative differences were used to determine the agreement between the two methods. Bland–Altman analyses revealed that the continuous glucose monitoring devices had proportional bias (*R* = 0.028, *p* < 0.001) and a mean bias of −0.048 mmol/L, and device measurements were more variable as glucose concentrations increased. Ninety-nine per cent of paired values were in Zones A and B of the Parkes Error Grid plot, and there was an overall mean absolute relative difference of 16.2% (±15.8%). There was variability in the continuous glucose monitoring devices, and this variability was higher when glucose concentrations were higher. If researchers were to use continuous glucose monitoring devices to measure glucose concentrations during an oral glucose tolerance test in healthy participants, this variability would need to be considered.

## 1. Introduction

The accurate measurement of glucose concentrations is critical in research where the impact of behaviours or psychological states on glucose concentrations is of interest. Traditionally, measuring glucose concentrations has been conducted by drawing blood from participants [1], but it can also be achieved with the use of continuous glucose monitoring devices. Continuous glucose monitoring devices measure glucose concentrations in interstitial fluid. Due to the differing glucose dynamics in interstitial fluid and blood plasma, there is a need for these devices to be validated. The devices have been validated previously for their use in clinical settings, e.g., [2,3], but they have not been validated for use during an oral glucose tolerance test in healthy individuals—a research area where they have the potential to be a valuable tool [4].

Glucose concentrations derived from continuous glucose monitoring devices show high levels of accuracy in patients with diabetes mellitus [5,6,7]. An analysis of continuous glucose monitor accuracy in patients with type 1 and type 2 diabetes was conducted by Bailey et al. [8]. Ninety participants wore continuous glucose monitoring devices for six days. On days one, three and six, blood samples were collected every 5 to 15 min (for 12 h), from which plasma glucose concentrations were measured. The devices had an overall mean absolute relative difference of 13.6%, which remained stable from day one to day six. That is, the device readings only differed from the plasma glucose concentrations by 13.6%. Consensus error grid analysis [9] also indicated that over 98% of readings from the devices were accurate enough that they would result in correct treatment decisions [8]. Device accuracy was not measured with Bland–Altman plots, and therefore the bias of one method of measurement over the other was not reported. This investigation by Bailey et al. focused on the use of continuous glucose monitoring devices in metabolically unhealthy participants (with type 1 or type 2 diabetes) [8]. Glucose dynamics can vary between metabolically healthy and metabolically unhealthy individuals [10,11]. Continuous glucose monitoring device accuracy is affected by glucose dynamics [12], and thus, the validation study conducted by Bailey et al. may not generalise to metabolically healthy individuals.

Continuous glucose monitoring devices are increasingly being used in research settings where the impact of different interventions on the glucose response in healthy participants are of interest, i.e. [13,14,15,16,17,18,19]. Instead of drawing blood samples or conducting finger pricks to determine glucose concentrations, continuous glucose monitoring devices can be used. For example, the devices have been used during an oral glucose tolerance test to observe the impact of sleep restriction on glucose tolerance [16]. They have also been used to measure the impact of sleep stages on glucose concentrations while participants were asleep [20]. The data sets in both studies were achieved without drawing venous or capillary samples from participants. This may have been advantageous as there was no limit to how many samples could safely be taken overnight, providing a rich data set. Using these devices could also mitigate the potential complications associated with drawing venous samples, such as the potential psychological distress to participants associated with needle insertion [21,22]. This advantage would be critical for researchers interested in the impact of psychological states such as stress or emotional arousal on glucose concentrations, e.g., [23,24]. Further adding to their usability in research protocols is that data can be stored on the devices while participants are not in contact with researchers (e.g., at home or work) for multiple days.

Despite their more frequent use in research settings, continuous glucose monitoring devices have not yet been validated for use during an oral glucose tolerance test in metabolically healthy participants. The oral glucose tolerance test is a standard test used to diagnose type 2 diabetes and measure glucose tolerance [25]. It is also commonly used in research protocols that aim to determine the influence of an independent variable on glucose tolerance, e.g., [26,27,28]. The test requires an individual to consume a 300 mL water-based drink containing 75 g of glucose. Blood samples are then collected at pre-determined intervals in the subsequent two or three hours. Glucose concentrations from the blood samples are used to determine the body’s response to the glucose drink, i.e., glucose tolerance [29]. Continuous glucose monitoring devices could be used to measure glucose concentrations during the oral glucose tolerance test instead of collecting venous samples. This would allow researchers to measure the glucose response resulting from various behavioural (e.g., food choice) or psychological (e.g., the Trier Social Stress Test) interventions without the need to collect blood from participants.

The primary aim of this research is to validate a continuous glucose monitoring device for use during an oral glucose tolerance test in healthy young adults. Bland–Altman plots will be used to compare glucose values from continuous glucose monitoring devices with those determined from blood plasma collected during an oral glucose tolerance test. A decrease in fasting glucose levels by 1 mmol/L is associated with a 58% reduction in risk of developing type two diabetes [28]. Therefore, a bias equal to or below 0.99 mmol/L will be considered an acceptable level of accuracy.

## 2. Materials and Methods

### 2.1. Participants

Flyers advertising the study were placed at backpacker hostels and University sites in Adelaide, South Australia, Australia. Flyers were also posted to the free online advertising service www.gumtree.com.au (accessed on 16 April 2018) and on the social media service www.facebook.com (accessed on 16 April 2018). Potential participants responded to these advertisements via email or phone call to a researcher and were provided information about the study. Potential participants then completed a health questionnaire and were invited to the laboratory for a screening interview.

One hundred and twenty-four healthy young males were recruited to participate in this study. Their average (±SD) age was 22.9 ± 3.7 years, and the average body mass index was 22.8 ± 2.1 kg/m². Participants were non-smokers and did not suffer from any metabolic disorders. All participants had fasting glucose concentrations below 5.5 mmol/L. Recruitment was limited to male participants to avoid the potential influence of female hormonal cycles on other outcome variables of interest in the wider project (reported elsewhere). The project was conducted in accordance with the Declaration of Helsinki and the protocol approved by the Central Queensland University Ethics Committee (H14/11-246). Participants provided signed, informed consent prior to participation.

### 2.2. Laboratory Setting

Participants lived in the sleep laboratory at the Appleton Institute for Behavioural Science, Central Queensland University in South Australia, Australia. The laboratory could accommodate six participants concurrently and contained six bedrooms, living rooms and bathrooms. It contained a kitchen, a communal dining room and an examination room that could seat six participants.

### 2.3. Protocol

**Figure 1:**
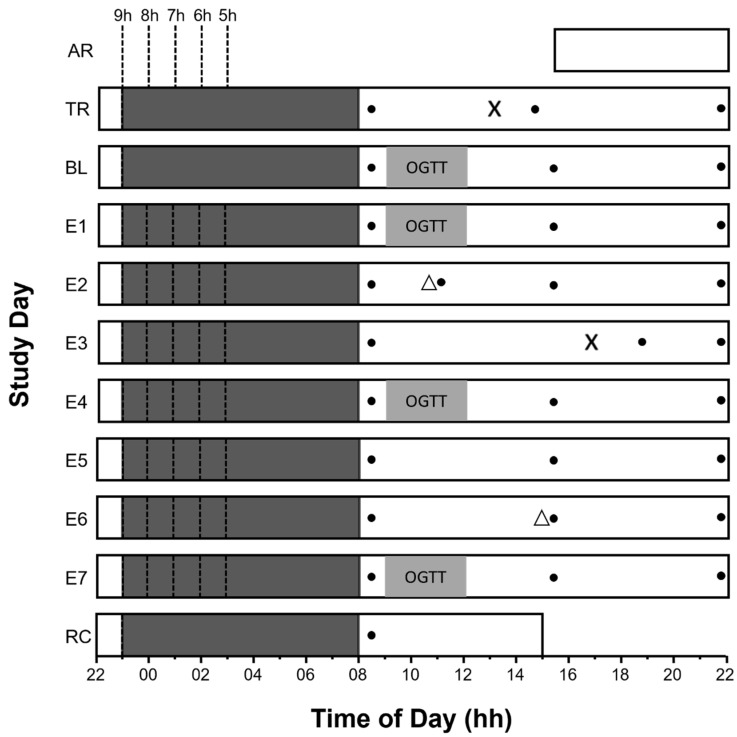
Study Protocol. A pictorial representation of the study protocol. The x-axis indicates the time of day from 22:00 h to 22:00 h. AR = arrival day, TR = training day, BL= baseline day. E1 to E7 = Experimental days 1 to 7, and RC = recovery day. The vertical dashed lines indicate bedtimes, and the solid dark grey horizontal bars represent time in bed. The X symbol indicates when the first and second sensors were inserted, and the solid black circles represent capillary blood testing times for device calibration. Triangles represent sensor restart times. Sensor age (hours since insertion) at 09:00 h is indicated on the right-hand side of the diagram. NS indicates no sensor was present at 09:00 h. The letters OGTT indicate when the oral glucose tolerance tests occurred.

The data presented here were collected as part of a larger project investigating the influence of time in bed on glucose tolerance. Data collection occurred between January 2015 and April 2018. Participants arrived at the laboratory on the arrival day (AR, Figure 1) at 16:00h, where they remained for ten nights. They were not permitted to leave until the conclusion of the study (unless they withdrew their participation). Time in bed was from 23:00 h to 08:00 h on nights one and two (TR and BL, Figure 1). On the following seven nights, time in bed ranged from five to nine hours, wake times remained fixed in all conditions (08:00 h), but bedtimes varied (Figure 1). Participants consumed breakfast (08:30 h), lunch (12:30 h), an afternoon snack (15:15 h), dinner (19:00 h) and an evening snack (20:15 h) on all days except BL, E1, E4 and E7. On BL, E1, E4 and E7, participants did not consume breakfast in order to fast prior to the oral glucose tolerance test.

Between 13:00 h and 14:00 h on the training day, researchers inserted glucose sensors into participants. The sensors were inserted approximately five centimetres to the left/right and five centimetres down from the naval. Approximately two hours later, the devices were calibrated using a capillary blood sample. The sensor was ‘restarted’ at 11:00 h on E2 to ensure it continued to operate beyond 72 h, as per the manufacturer’s recommendations (Figure 1). Between 16:00 h and 17:00 h on E3, researchers replaced the sensors with new ones on the opposing side to where the first sensor was inserted. The new sensor was restarted at 15:00 h on E6 to ensure it continued to operate beyond 72 h (Figure 1). Capillary blood testing for device calibration was conducted by researchers each day at 08:30 h, 15:30 h and 22:30 h.

### 2.4. Oral Glucose Tolerance Test

Three-hour oral glucose tolerance tests were conducted on BL, E1, E4 and E7 (Figure 1). Participants entered the examination room at 08:35 h, and a 16-gauge AV fistula needle set (SysLoc 16Gx1” Back Eye 30 cm with Clamp; JMS Singapore Pty Ltd., Singapore) was inserted into the deep muscle branch of an antecubital vein. The needle set was filled with heparinised saline (Heparin Sodium 1000IU, Sodium Chloride 0.9%; Baxter, Toongabbie, Sydney, Australia), sealed with a three-way stopcock (Discofix C; B. Braun, Melsungen, Hesse, Germany) and taped to the skin. Once all needles had been inserted and were patent (approximately 09:00 h), participants consumed a glucose bolus; 75-g of glucose and 24-g of sodium dissolved in water (Carbotest; Thermo Fisher Scientific, Scoresby, VIC, Australia). Blood samples were collected at 0, 10, 20, 30, 60, 90, 120, 150 and 180 min. Following the collection of each whole blood sample, 2 mL of heparinised saline was introduced into the vein to maintain patency, which was discarded prior to the collection of each sample. At each sampling time point, 4 mL of whole blood was collected into a syringe (10 mL Luer Eccentric; Terumo, Leuven, Belgium) and immediately transferred into a storage tube (6ml FX Sodium Fluoride/Potassium Oxalate; Greiner Bio-One, Kremsmünster, Austria). Samples were centrifuged (Universal 320R, Hettich, Tuttlingen, Germany) for 10 min at 4 °C within 30 min of collection. Aliquots (1500 μL) were stored at −20 °C until they were analysed for the plasma concentration of glucose (Konelab, Thermo Electron Corporation, Louisville, KY, USA) using a commercial enzymatic kit (Thermo Fisher Scientific, Melbourne, Australia).

### 2.5. Continuous Glucose Monitoring Device

Interstitial glucose concentrations were assessed using a continuous glucose monitoring device (Medtronic Guardian; Medtronic, Northridge, CA, USA). The device consists of a sensor, a transmitter and a recording device. The sensor (Enlite Glucose Sensor; Medtronic, Northridge, CA, USA) is a thin (31-gauge), short (8.5 mm) substrate with electrode surfaces to detect glucose concentrations. The Enlite sensor was chosen as it is compatible with the Medtronic Guardian system. Only one brand of sensor was used to allow for comparisons in the glucose response between groups (data reported elsewhere). The sensor is inserted into the skin using an introducer needle which is discarded after insertion. The transmitter is a small (approximately 2 cm in diameter) flat, round device which is attached to the exposed part of the sensor and covered with a waterproof dressing. The transmitter sends radio signals depicting interstitial glucose concentrations to the recording device, where they are recorded in 5-min epochs.

### 2.6. Capillary Blood Sampling Device

Capillary blood samples to calibrate the monitors were obtained using a 28-gauge lancet with a penetration depth of 1.6 mm (Haemolance Plus^®^ Micro Flow; Ozorkow, Poland). Samples were analysed with a portable glucose meter (Accu-Chek^®^ Performa; Manaheim, Baden-Württemberg, Germany) in combination with glucose test strips (Accu-Chek^®^ Performa test strips; Manaheim, Baden-Württemberg, Germany). The meter meets the accuracy standards outlined by ISO 15197:2013, with 87–97% of readings within 5 mg/dL when glucose concentrations were below 100 mg/dL and 71 to 77% of readings within 5 mg/dL when glucose concentrations were above 100 mg/L [30].

### 2.7. Analysis

Agreement between glucose concentrations from continuous glucose monitoring devices and glucose concentrations from blood plasma was assessed using the limits of agreement method for repeated measurements, referred to as Bland–Altman plots [30]. The difference between each pair of measurements was plotted against the mean of the corresponding pairs of measurements. The mean difference between the two measurements (the bias) and the 95% limits of agreement (bias ± SD) were also plotted. All data sets were tested for heteroscedasticity using the Breusch–Pagan test and for proportional bias using ordinary least squares regression. The data was heteroscedastic and proportional bias was present when all data points were combined on BL and E1; therefore, the limits of agreement were adjusted accordingly [31,32]. Device point accuracy was assessed using the mean absolute relative difference—the average of the relative differences for all paired points [33] and the Parkes error grid analysis [34].

All glucose values from continuous glucose monitoring devices were matched to the corresponding glucose concentrations obtained during the oral glucose tolerance tests using Microsoft Excel (Microsoft Office 365 ProPlus 2019, Version 1812). The continuous glucose monitoring devices stored glucose concentrations in 5-min epochs, and a single data point was representative of the average glucose concentration of the previous five minutes. Therefore, glucose values obtained from blood plasma samples within the preceding five minutes of a reading from a continuous glucose monitoring device were considered to be of the same time point [6].

Glucose concentrations in interstitial fluid appear later than they do in blood plasma by at least eight minutes [35]. This time delay can vary depending on the glucose concentration and the rate of change—in some cases, this time delay can be up to 40 min, particularly when glucose is falling at a rapid rate [12]. To determine the duration of the delay in our data set, we matched the clock time that blood plasma samples were drawn with the value from the continuous glucose monitoring device that occurred at the end of the next five-minute epoch. The time at which the interstitial glucose concentration was recorded was then adjusted (delayed) by 5, 10, 15, 20 and 25 min. Spearman rank order correlations were then used to determine which delay (in interstitial glucose concentrations) correlated highest with blood glucose concentrations from blood plasma.

## 3. Results

### 3.1. Participants, Sample and Missing Data

A total of 124 participants were recruited to this study, and eight participants withdrew. The participants who withdrew did not cite reasons for their withdrawal. The remaining 116 participants had a mean (±SD) age and body mass index of 22.9 ± 3.7 years and 22.9 ± 1.97 kg/m², respectively.

A total of 3315 glucose values from blood plasma were paired with glucose values from continuous glucose monitoring devices. During the oral glucose tolerance tests, the total number of possible blood samples to be collected was 4960 (124 participants, 4 glucose tolerance tests, 10 samples per test). Of the 4960 possible samples, 299 samples were not collected due to loss of patency of the fistula needle (n = 34 samples) or the fistula being removed due to the participant feeling lightheaded (n = 4 participants). Data from 25 participants were not assayed due to missing data from their baseline oral glucose tolerance tests (n = 17 participants, n = 612 samples) or participant withdrawal (n = 8 participants, n = 261 samples). These data sets were excluded because they were considered incomplete, and as such, they did not benefit other aims of the project (reported elsewhere).

The remaining missing data points (n = 238 data points) were due to the cessation of recording of the glucose monitoring devices. This happened because the sensor became detached from the skin on the morning of the oral glucose tolerance test (n = 36 data points), or the pager was left out of range of the sensor for longer than five minutes, and the radio signal was lost (n = 198 data points).

### 3.2. Correlations between Plasma Glucose Concentrations and Interstitial Glucose Concentrations

Data for plasma and interstitial glucose concentrations (at all delay times) were not normally distributed (all *p* values < 0.05), thus, Spearman rank order correlations were used. The highest correlation between plasma glucose concentrations and interstitial glucose concentrations occurred with a 15-min delay (Table 1). Therefore, a 15-min delay was applied to the glucose values obtained from the continuous glucose monitoring devices for all subsequent analyses.

### 3.3. Bland–Altman Plots

Shapiro–Wilk tests were used to determine if the differences between the blood glucose values from plasma and the glucose values from continuous glucose monitoring devices were normally distributed [36]. The data sets from baseline, experimental day one, four and seven were not normally distributed. However, Bland–Altman plots are robust to violations of normality, therefore, the parametric approach to Bland–Altman plots was applied [36].

**Figure 2:**
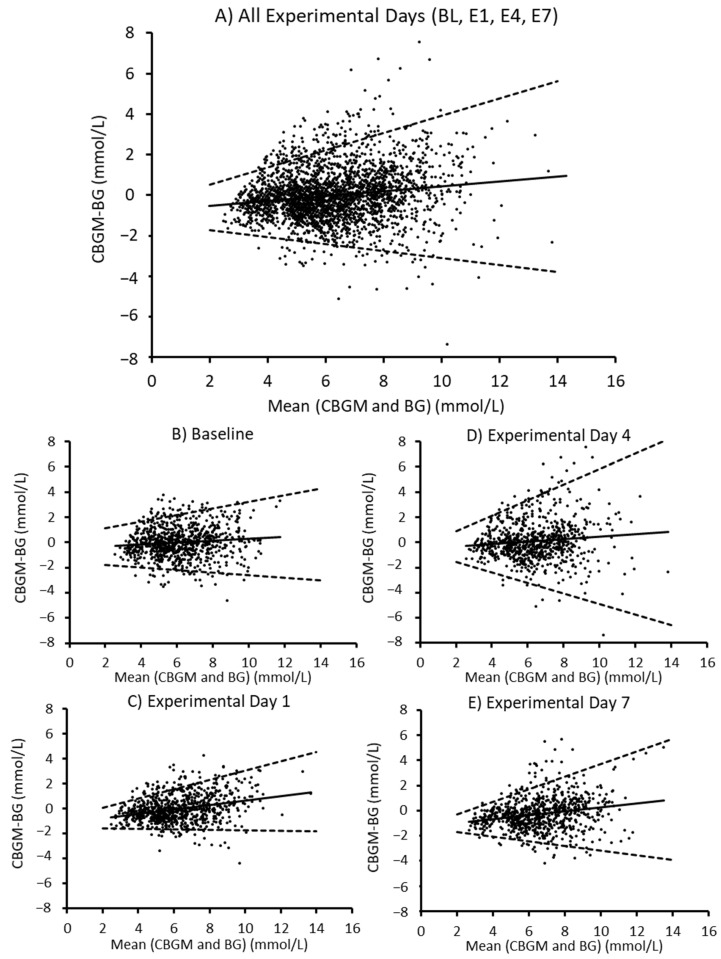
Comparison of blood plasma glucose concentration and continuous glucose monitoring device. Bland–Altman plots comparing blood plasma with continuous glucose monitoring devices for all data points combined (panel **A**), baseline day (panel **B**), experimental day 1 (panel **C**), experimental day 4 (panel **D**) and experimental day 7 (panel **E**). The x-axes represent the mean of the values obtained from blood plasma and continuous glucose monitoring devices, and the y-axis represents the difference (mmol/L) between the values. Positive values indicate an overestimation by continuous glucose monitoring devices, and negative values indicate an underestimation in comparison with blood plasma. Solid sloped lines indicate the mean bias from continuous glucose monitoring devices, and dashed lines indicate the 95% limits of agreement (±1.96 SDs). CBGM—continuous blood glucose monitoring, BG—blood glucose levels derived from plasma.

**Figure A1:**
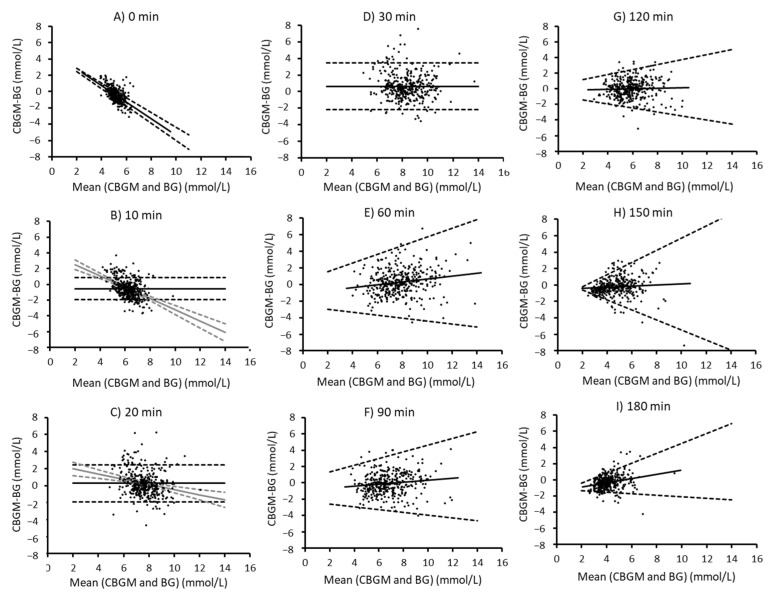
Bland–Altman plots stratified for sample time points during the oral glucose tolerance test.

There was proportional bias for all data points combined (*R* = 0.028, *p* < 0.001), and the mean bias was −0.048 mmol/L. For all data points (Figure 2, panel A), when the mean of continuous glucose monitoring values and blood plasma values were 2 and 14 mmol/L, the upper limits of agreement were 0.53 and 5.62mmol/L, respectively, and the lower limits of agreement were −1.71 and −3.79 mmol/L, respectively. There was proportional bias on days BL (*R* = 0.012, *p* = 0.001), E1 (*R* = 0.08, *p* < 0.001), E4 (*R* = 0.012, *p* = 0.002) and E7 (*R* = 0.053, *p* < 0.001). That is, continuous glucose monitoring devices progressively overestimated glucose concentrations as glucose concentrations increased. The mean bias was −0.05 mmol/L on BL, −0.01 mmol/L on E1, 0.10 mmol/L on E4 and −0.28 mmol/L on E7 (Figure 2). Bland–Altman plots stratified for sample time points during the oral glucose tolerance test are provided in Appendix A (Figure A1).

### 3.4. Mean Absolute Relative Difference

The mean (±SD) absolute relative difference was 16.2% (±15.8%) for all paired data points, 16.3% (±14.9%) on BL, 13.7% (±11.9%) on E1, 19.3% (±20.9%) on E4 and 15.5% (±14.1%) on E7. The mean absolute relative difference was significantly different across days χ²(3) = 33.09, *p* < 0.001. Wilcoxon signed rank tests (Bonferroni correction, *p* value of 0.008) indicated it was higher on BL than on E1 (*T* = 150815, *r =* −0.07), higher on E4 than on E1 (*T* = 118687, *r* = −0.13), higher on E7 than on E1 (*T* = 135065, *r* = −0.08) and higher on E4 than on BL (*T* = 133028, *r* −0.07).

### 3.5. Error Grid Analysis

**Figure 3:**
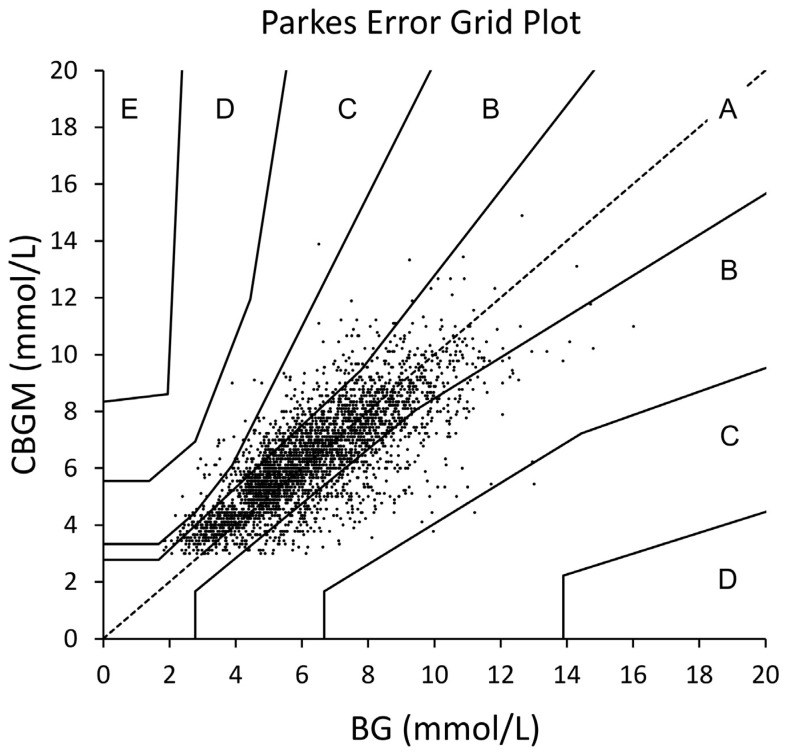
Parks Error Grid Plot. The x-axis represents blood glucose concentrations derived from blood plasma (BG) and the y-axis represents glucose concentrations derived from continuous glucose monitoring devices (CBGM) in mmol/L. The solid, fanned lines represent the five zones of the error grid plot from Zone A to Zone E.

The Parkes Error Grid analysis for data from all experimental days indicated that 77.89% of values were in Zone A, 21.15% of values were in Zone B and 0.96% of values were in Zone C. None of the values were in Zones D or E (Figure 3).

## 4. Discussion

We assessed the accuracy of continuous glucose monitoring devices when measuring glucose concentrations in healthy participants. The devices have previously been validated for use in clinical settings in patients with diabetes mellitus [37], but they are increasingly being used in laboratory settings to measure the effects of different behaviours on glucose concentrations in healthy individuals. We compared the accuracy of continuous glucose monitoring devices at measuring blood glucose concentrations to those derived from blood plasma during a 3-hour oral glucose tolerance test.

Agreement between the two methods was assessed using the Bland–Altman method for repeated measurements [32]. The overall mean bias (across all days of the study) was below 0.05 mmol/L, and on the individual days (BL1, E1, E4, E7) it was below 0.99 mmol/L. This is lower than what would be considered a meaningful change in glucose concentrations [28]. It is also consistent with previous research where the mean bias between continuous glucose monitoring devices and blood plasma glucose concentrations was lower than 5% [6]. There was also proportional bias, such that, as glucose concentrations increased, continuous glucose monitoring devices overestimated glucose concentrations. The slope of this line was low for all experimental days combined (Figure 1, panel A), and at 14 mmol/L, continuous glucose monitoring devices would overestimate glucose concentrations by 0.99 mmol/L. We also considered the orientation and distance of the 95% limits of agreement. The limits of agreement were expressed in a v-shape to account for the heteroscedasticity of the data [32]. Thus, as glucose concentrations increased, so did the variability of continuous glucose monitoring devices. This pattern was consistent across all days of measurement and within different time points of the oral glucose tolerance test (Appendix A, Figure A1).

For the entire data set, the mean absolute relative difference (MARD) was 16.2% (±15.8%). Similar values have been reported in other studies using the same brand of sensor (the Enlite sensor from Medtronic), for example, 16.6% (±13.5%) from Kropff et al. [6] and 16.7% (±19.3%) from Freckmann et al. [38]. Bailey et al., however, reported an overall MARD of 13.63% (±13.9%), slightly lower than our findings and those of others [8]. The reason for this lower MARD may be due to the participants spending longer time periods in slow rates of glucose change [8]. Indeed, higher rates of glucose change can result in a higher MARD [39]. A MARD value of 10% is considered the cut-point for the clinical use of continuous glucose monitoring devices in patients at risk of hypo or hyperglycaemia [40]. In the present study, the lowest MARD value of 13.1% (±11.9%) was found on experimental day one. Therefore, the MARD values achieved in the present dataset did not meet the highest standard of clinical accuracy [40]. This was possibly due to the higher rate of glucose change that would have occurred during the oral glucose tolerance tests [41]. Indeed, the fasting MARD values (Appendix A) were lower than those recorded during the oral glucose tolerance test.

Both the limits of agreement and the mean absolute relative differences varied on different days of measurement. These variations may reflect sensor age. For example, the MARD was highest, and the limits of agreement were widest when the sensor age was 17 h (Figure 1). It may be that after 17 h of wear, the sensor was not yet working optimally due to tissue trauma associated with insertion [42]. Indeed, sensor accuracy has previously been shown to improve after the first 24 h of wear [43], possibly due to the insertion area recovering from the trauma.

We also analysed our data for point accuracy using the Parkes error grid plot. Error grid analysis allows for the determination of clinical accuracy of continuous glucose monitoring devices while accounting for glucose value, direction and rate of change [44]. The zones for type 1 diabetes were used as they are more conservatively spaced. Over 99% of values were in Zone A (77.89%) and B (21.14%) of the plot. Values in Zone A are considered clinically accurate and are within 20% of the reference blood glucose values. Treatment decisions based on values in Zone B are considered to have little or no effect on clinical outcomes [45]. We did not induce hypo or hyperglycaemia in our participants, and therefore, the point accuracy reported here represents accuracy when blood glucose concentrations are within a normal range. Previous investigations have found up to 86% of values in Zone A [8]. The lower point accuracy observed in our study compared to Bailey et al. (2014) may be due to the potential higher rate of glucose change in our study (as previously discussed).

These results indicate that using continuous glucose monitoring devices to measure glucose concentrations in healthy participants during an oral glucose tolerance may be an alternative to sampling blood plasma concentrations. The devices have already been used adeptly in sleep research to monitor the impact of sleep restriction [15,16] and sleep stages [20] on glucose concentrations. The devices could also be used to measure the impact of various psychological or biobehavioral interventions on glucose concentrations. For example, researchers may wish to determine the impact of emotionally arousing images [23] or psychological stress [46] on glucose concentrations. This could be achieved with continuous glucose monitoring devices without causing psychological distress to participants through repeated capillary finger pricks [47] or needle insertion [21]. If researchers wish to use an oral glucose tolerance test to measure glucose concentrations following a behavioural intervention, they should be aware of the variability associated with continuous glucose monitoring devices.

One limitation of the present research was the use of only one brand of glucose sensor—the ENLITE sensor from Medtronic (Enlite Glucose sensor; Medtronic, Northridge, CA, USA). Future research may investigate the performance of other sensors under similar conditions. Additionally, accuracy was only assessed during an oral glucose tolerance test and not while participants had the freedom to choose what and when they ate, during exercise or while sleeping. Another factor that may have influenced the accuracy of the sensors was the use of capillary samples for calibration. To minimise error associated with this, capillary samples were collected in a standardised manner by trained research staff and samples were not collected during times of rapid glucose change but rather after periods of fasting [48]. Finally, our sample only included young, adult male participants. Including female participants and expanding the age range to greater than 30 years would increase the external validity of the findings.

## 5. Conclusions

The bias of continuous glucose monitoring devices was proportional and below 0.99 mmol/L. There was measurement variability as indicated by the standard deviations of the mean absolute relative difference and the limits of agreement. Thus, if researchers were to rely solely on continuous glucose monitoring devices to assess glucose concentrations during an oral glucose tolerance test in healthy participants, awareness of the measurement variability would be required. However, sensor hardware and manufacturers’ supporting algorithms used to measure glucose concentrations are continually being upgraded and improved [49]. Therefore, their efficacy in such settings may improve in the future; however, more research to validate the updated devices will be required.

## Figures and Tables

**Table 1:** Spearman correlations between plasma blood glucose concentrations and interstitial glucose concentrations at different time delays (*n* = 116).

	Experimental Day				
Delay	BL	E1	E4	E7	All Days
+0 min	0.608 *	0.705 *	0.565 *	0.689 *	0.643 *
+5 min	0.676 *	0.797 *	0.649 *	0.78 *	0.726 *
+10 min	0.706 *	0.838 *	0.686 *	0.784 *	0.755 *
+15 min	0.756 *	0.842 *	0.682 *	0.8 *	0.771 *
+20 min	0.727 *	0.81 *	0.664 *	0.774 *	0.745 *
+25 min	0.679 *	0.763 *	0.626 *	0.729 *	0.701 *

Note. * all values significant at the *p* < 0.001 level. BL = baseline, E1= experimental day 1, E2 = experimental day 2, E4 = experimental day 4, E7 = experimental day 7. The time (minutes) in the first column indicates the number of minutes that the continuous glucose monitoring values were delayed by to achieve the correlation value in subsequent columns.

## Data Availability

The datasets generated from the study are available from the corresponding author on reasonable request.
